# Single-atom nanozymes shines diagnostics of gastrointestinal diseases

**DOI:** 10.1186/s12951-024-02569-3

**Published:** 2024-05-25

**Authors:** Sijia Hua, Xiulin Dong, Qiuxia Peng, Kun Zhang, Xiaofeng Zhang, Jianfeng Yang

**Affiliations:** 1 Zhejiang University of Chinese Medicine, No. 548 Binwen Road, Binjiang District, Hangzhou, 310053 Zhejiang China; 2 Department of Gastroenterology, School of Medicine, Affiliated Hangzhou First People’s Hospital, Westlake University, No. 261 Huansha Road, Hangzhou, 310006 Zhejiang China; 3 Department of Pharmacy and Central Laboratory, School of Medicine, Sichuan Academy of Medical Sciences, Sichuan Provincial People’s Hospital, University of Electronic Science and Technology of China, No. 32, West Second Section, First Ring Road, Chengdu, 610072 Sichuan People’s Republic of China

**Keywords:** Single-atom nanozymes, Catalytic regulation, Gastrointestinal diseases, Cancer therapy, Bioassay, Antibacterial

## Abstract

Various clinical symptoms of digestive system, such as infectious, inflammatory, and malignant disorders, have a profound impact on the quality of life and overall health of patients. Therefore, the chase for more potent medicines is both highly significant and urgent. Nanozymes, a novel class of nanomaterials, amalgamate the biological properties of nanomaterials with the catalytic activity of enzymes, and have been engineered for various biomedical applications, including complex gastrointestinal diseases (GI). Particularly, because of their distinctive metal coordination structure and ability to maximize atom use efficiency, single-atom nanozymes (SAzymes) with atomically scattered metal centers are becoming a more viable substitute for natural enzymes. Traditional nanozyme design strategies are no longer able to meet the current requirements for efficient and diverse SAzymes design due to the diversification and complexity of preparation processes. As a result, this review emphasizes the design concept and the synthesis strategy of SAzymes, and corresponding bioenzyme-like activities, such as superoxide dismutase (SOD), peroxidase (POD), oxidase (OXD), catalase (CAT), and glutathione peroxidase (GPx). Then the various application of SAzymes in GI illnesses are summarized, which should encourage further research into nanozymes to achieve better application characteristics.

**Graphical abstract:**

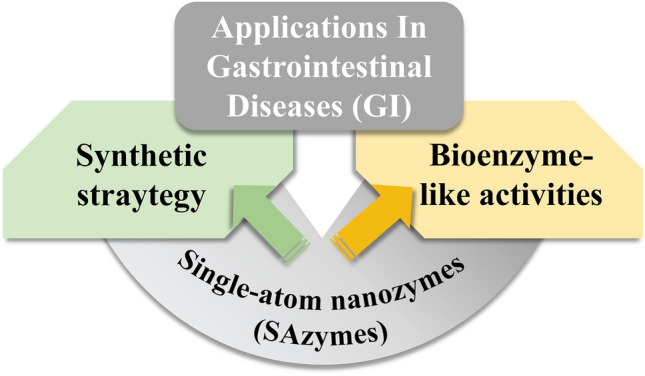

## Introduction

Hospitalizations for digestive system disorders have risen absolutely as a proportion of all hospitalizations over the past two decades, with the largest rises in disorders including, intestinal infections and pancreatitis, posing significant implications for the quality of life and overall health of patients [1, 2]. Tumors of the digestive system (e.g., liver cancer [3], pancreatic [4], and colorectal cancer [5]) also account for a large percentage of solid tumors. Despite the constant updating of therapeutic approaches, the incidence and mortality of certain diseases, such as gastric, esophageal, and colorectal cancers, are increasing annually [6]. Although significant progress has been made in the detection and treatment of digestive illnesses since the advent of endoscopic techniques [7], novel therapeutic approaches are still required to address the complexity and diversity of disease types. Catalytic nanomaterials known as nanozymes have features similar to those of enzymes and come in a variety of sizes, shapes, and surface arrangements. These artificial enzymes exhibit both great catalytic stability and high catalytic efficiency. It has been demonstrated that nanozymes possess the biocatalytic activity of naturally occurring enzymes, such as SOD [8–10], OXD [11, 12], POD [13], CAT [9, 14] and so on. The focus of nanozyme research is currently on single-atom nanozymes (SAzymes), a novel class of nanozymes with the benefits of high atom utilization, high catalytic activity, cheap production cost, and great selectivity. SAzymes are also gradually being developed for the treatment of digestive diseases, such as liver cancer and cirrhosis [15], pancreatitis [16], and the control of intestinal inflammation [17].

**Fig. 1:**
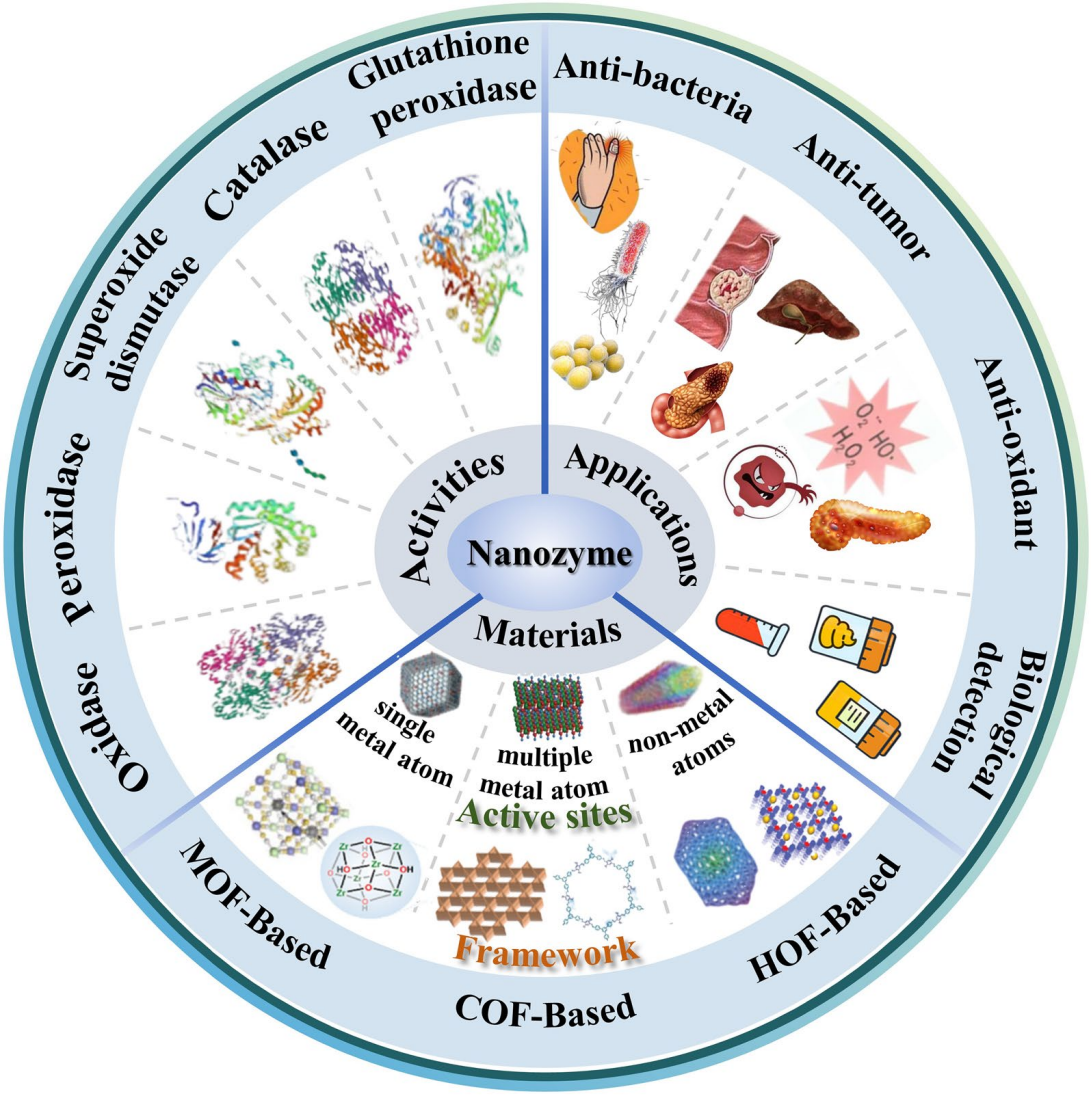
Schematic overview. Recent developments in nanozymes in terms of material preparation, enzyme-like catalytic activity and biological applications of nanozymes in Gastrointestinal (GI) diseases

Herein, an emphasis on their unprecedent design concepts to optimize dispersion, stability, and enzyme activity has been made. Based on it, we give a brief summary of the function of the biocatalytic-like activity of SAzymes and the corresponding application innovation of theme in the field of gastrointestinal disorders. Finally, in response to the problems and controversies of SAzymes in terms of bioactivity and biological applications, we point out the future opportunities and challenges of SAzymes and propose potential solutions and future directions to accelerate the development of SAzymes in biomedical fields (Fig. 1). Finally, an overview of the biological uses of SAzymes, along with issues and disagreements surrounding them, is provided. The outlook and challenges confronting SAzymes in the forthcoming period are also highlighted along with potential solutions and future pathways to expedite their development within the biomedical industry.

## Design principles and synthesis strategy of SAzymes

**Fig. 2:**
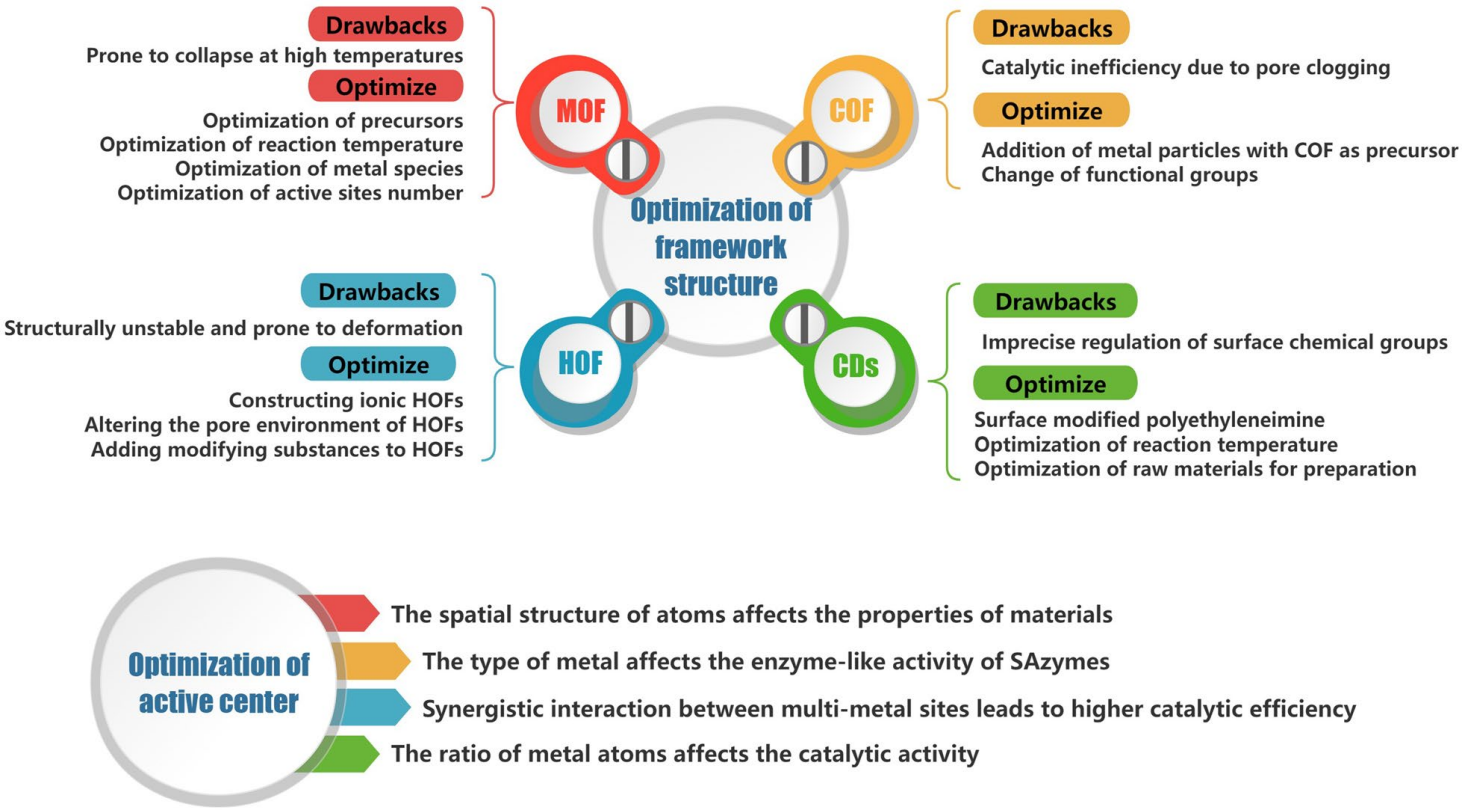
Optimization strategies for single-atom nanozymes at both framework structure and active center levels

The preparation of frameworks, such as metal–organic frameworks (MOFs), covalent organic frameworks (COFs), hydrogen-bonded organic frameworks (HOFs) frameworks with various active centers, has actually been linked to a wide range of multi-enzyme activities. Because of this, scientists have experimented with a number of design approaches in an effort to maximize the dispersion, stability, especially catalytic activity in recent years. This section focuses on the remarkable advancements made recently in engineering techniques for activity tuning, such as modifications to the SAzymes framework and optimizations for SAzymes active centers. Based on this, the particular synthesis processes and modalities of SAzymes are described in order to further analyze their synthesis strategy (Fig. 2).

### Optimization of framework structure

It is essential to use an appropriate supporting substrate while synthesizing SAzymes. This is because, as a result of aggregating during catalysis, instability in the framework may cause the core metal atom to become inactive. The atoms that comprise the framework have the ability to interact with the atom of the core metal, so influencing the metal’s activity and, consequently, the total catalytic activity of nanozymes. Consequently, in this section, we investigate how the activity of nanozymes is affected by the optimization of various kinds of framework materials. MOFs, COFs, and HOFs are examples of crystalline porous materials that typically have excellent porosity and structural composition designability. They are also thought to have controllable morphologies, modifiable backbones, flexible active site structures, tunable charge transfer pathways, and designable porosities [18, 19].

**Fig. 3:**
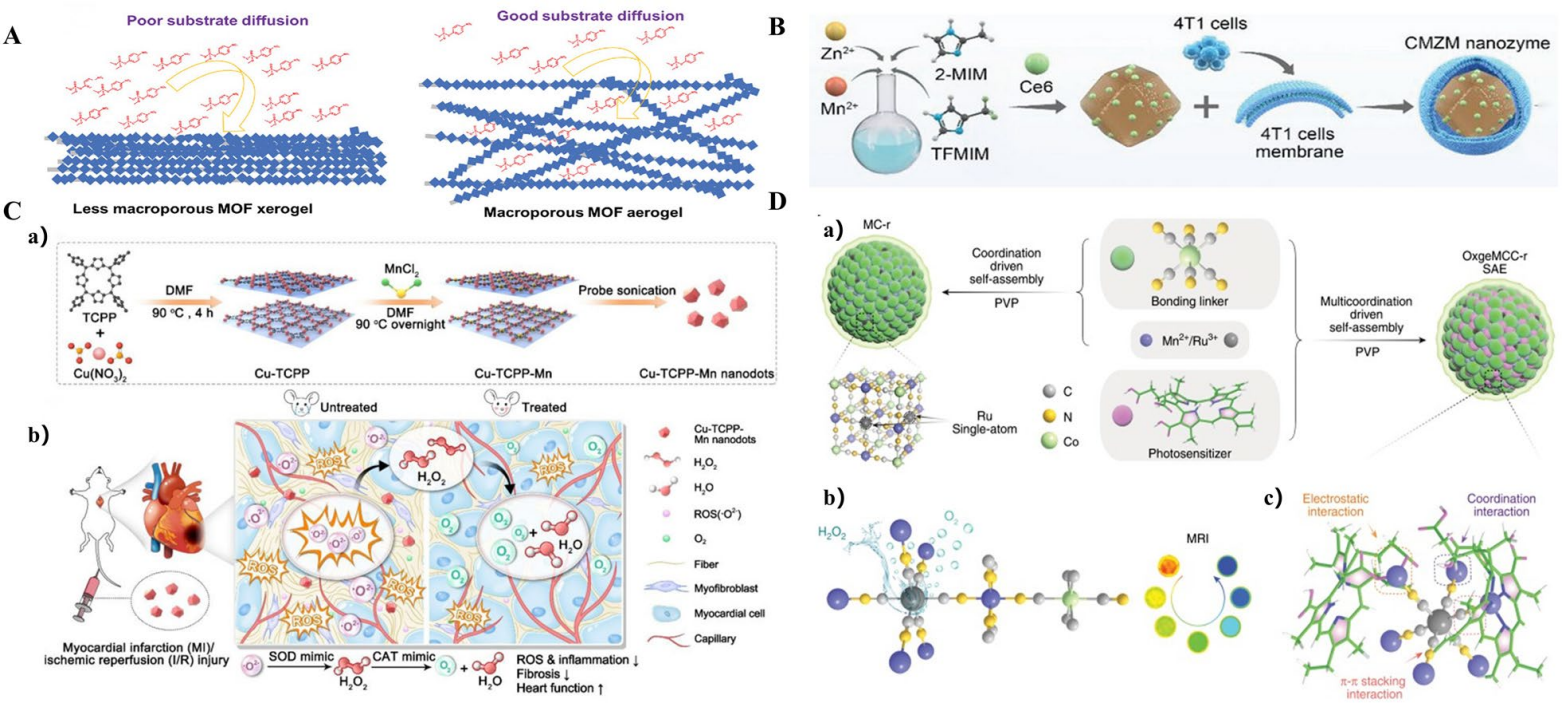
Optimization scheme for MOF structures.** A** Illustration of the differences in diffusion between the less macroporous MOF nanozyme xerogel (left) and the microporous MOF nanozyme aerogel (right). Reproduced with permission [21]. Copyright 2023. Wiley–VCH.** B** Illustration of CMZM preparation process. Reproduced with permission [14]. Copyright 2023. Wiley–VCH.** C** Schematic illustration of the design and synthesis of Cu-TCPP-Mn nanozyme for myocardial injury treatment. a) The bimetallic Cu-TCPP-Mn nanozyme was fabricated by embedding manganese and copper into the porphyrin via solvothermal method, followed by sonication into small MOF nanodots. b) Cu-TCPP-Mn nanozyme retained cascade activity that has been shown to scavenge ROS, inhibit inflammation, reduce myocardium fibrosis and promote constructive remodeling and vascularization in MI and I/R injury animal models. Reproduced with permission [22]. Copyright 2023. Ivyspring International Publisher.** D** Schematic illustration of OxgeMCC-r. a) Schematic illustration of OxgeMCC-r. OxgeMCC-r consists of catalytically active single-atom Ru site anchored in MCC with outer PVP protection layer. b) Partial molecular structure of OxgeMCC-r with active single-atom Ru site serving as catalase-like nanozyme for oxygen generation. c) Multicomponent coordination interactions within the OxgeMCC-r SAE. Reproduced with permission [28]. Copyright 2020. Springer Nature

MOFs are unique because of their ability to fine-tune pore structure and their variety in design. They are created by coordinating metal ions with organic ligands [20]. They are typically made using a one-pot self-assembly method that creates metal-containing nodes on the spot. Ma et al. [21] introduced Zr-MOF nanozymatic coatings into natural bacterial cellulose (BC) nanofibers, and this design enabled the nanozymes to have multilayered macro–micro pores, which led to the full exposure of catalytic active sites and exhibited excellent enzyme-mimicking catalytic activity (Fig. 3A). The same is true for CMZM, which has multi-enzymatic activity and can be utilized to reverse immunosuppressive TME. Additionally, the enzyme-like activities of CMZM improve the effectiveness of multimodal imaging-guided CDT and PDT treatments [14] (Fig. 3B). The bimetallic MOF pathway has been applied to the optimization of MOFs. The Cu, Mn bimetallic nanozymes (Cu-TCPP-Mn) prepared from MOFs have combined high catalytic properties for SOD and CAT, and can synergistically scavenge ROS (Fig. 3C) [22]. Another crystalline porous materials, COF, constructed through covalently linked organic units, show excellent properties for synthesizing SAzymes [23–25]. The one-dimensional iron porphyrin covalent organic skeleton (COF-CNT) coated on carbon nanotubes nanozymes were able to produce reactive oxygen species (ROS). Furthermore, the peroxidase-like activity of COF-CNT was much enhanced in the presence of an electric field, suggesting that the COF framework had a discernible impact on the enzymatic activity of the nanozymes [26]. When loaded with enzymes, pore congestion or partial clogging prevents COF from being used. HOFs are less stable than MOFs and COFs because they rely on hydrogen bonding interactions to stabilize their structure. However, because hydrogen bonding is typically weaker than ionic or covalent bonding, HOFs have an additional deformability that can be used to create flexible porous HOF materials [27].

**Fig. 4:**
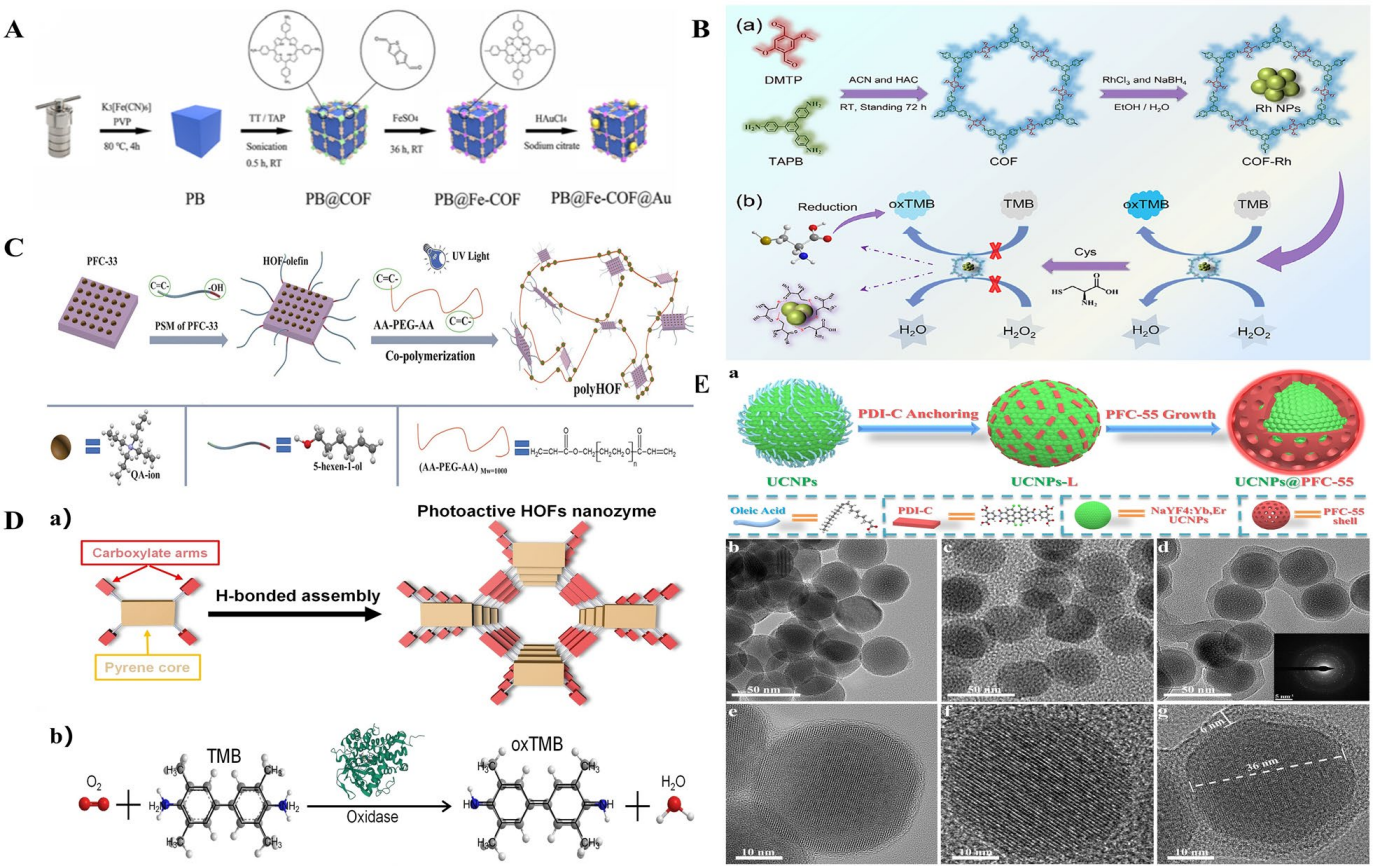
Optimization scheme for COF and HOF structures.** A** Procedure for the Synthesis of PB@Fe-COF@Au. Reproduced with permission [30]. Copyright 2023. American Chemical Society.** B** Schematic illustration showing a) the synthesis of COF and COF-Rh, and b the strategy for Cys detection based on regulating the POD-like activity of COF-Rh. Reproduced with permission [33]. Copyright 2023. Elsevier.** C** Preparation of polyHOF by PSM followed by polymerization. Reproduced with permission [35]. Copyright 2020. Wiley–VCH.** D** a) Schematic of hydrogen bonding assembly of HOFs nanozymes. b The oxidase-like catalysis using TMB as the substrate. The colors used are: red for Oatom; blue for Natom; gray for Catom; white for Hatom. Reproduced with permission [36]. Copyright 2023. Wiley–VCH.** E** Fabrication of core–shell UCNPs@PFC-55. a) Fabrication of core–shell UCNPs@PFC-55. b, e TEM images of as-synthesized oleate-stabilized β-NaYF4:Yb,Er UCNPs; c, f PDI-C anchoring UCNPs-L; d, g the final products UCNPs@PFC-55 with inorganic UCNPs “cores” and organic PFC-55 “shells”. Reproduced with permission [38]. Copyright 2021. Wiley–VCH

Increasing the catalytic activity of SAzymes through optimization of these frameworks has become a major area of research interest. An essential technique for creating N-doped carbon materials for stabilizing and spreading substrates supported by enzymes is the pyrolysis of MOFs. The nanozymes prepared either by doping Ru into the framework [28] or by encapsulating ferritin in ZIF-8 [29] have excellent stability and catalytic activity (Fig. 3D). It is also possible to tune the Fe–N coordination by optimizing the precursor type and pyrolysis temperature. Triple peroxidase-like activity and triple catalytic sites were added by Li et al. [30] to complement the synthesis of a unique core–shell nanocomposite, Prussian blue@Fe-covalent organic framework@Au (PB@Fe-COF@Au) (Fig. 4A). On the other hand, the strategy of synthesizing porous N-doped carbon nano-enzymes using COF as a precursor increased the exposure of the catalytic site and exhibited stronger peroxidase-like activity [31]. Innovatively grown platinum nanoparticles and ultramicro rhodium nanoparticles on the surface of COF NPs, Gao et al. [32] and Zhang et al. [33], respectively, served as catalase mimics in situ. The optimized nanozymes demonstrated high affinity for the catalytic substrates and excellent peroxidase-mimicking activity (Fig. 4B). In addition, the functional groups in the COF can act as Lewis acid–base sites within the porous skeleton to mimic the functions of amino acid residues and tailor the pore microenvironment around the active center, thus enhancing the catalytic activity of MOF-based nanozymes [34]. Constructing ionic HOFs is one of the strategies for HOF framework optimization. PFC-33 [35], the first anionic HOF synthesized, exhibits synergistic photodynamic and chemical antimicrobial efficiencies (Fig. 4C). Based on this, Tong et al. [36] created three light-responsive HOFs nanomaterials with various pore structures. Next, they mimicked light-responsive oxidative enzymes using structurally well-defined hydrogen-bonded organic frameworks (HOFs), and by creating isostructured HOFs, they were able to demonstrate the importance of pore environments in mediating the activity of oxidative enzyme-like enzymes. This demonstrated that, in addition to active centers, pore environments have a significant impact on the activity of the nanozymes (Fig. 4D). They suggested that the difference in pore channels could regulate the activity of isotope-like oxidase in structured HOFs. Ionic HOFs with anionic or cationic backbones were designed and synthesized to attract noncovalently bonded counterions in the pore channels. Currently, another optimization strategy for HOF frameworks is to add modifying substances to HOFs to improve the porosity and photodynamic efficiency. Yin et al. [37] performed surface modification by adding rabies virus glycoprotein (RVG)29. Not coincidentally, the final shell nanostructures were created by Liu et al. [38] utilizing a progressive ligand grafting technique (Fig. 4E). It is evident that altering the morphology or spatial arrangement of HOFs affects the catalytic activity of the nanocomplexes built using them as frameworks; this may be due to the catalytic sites being completely exposed through physical or chemical mechanisms. Similar to the dual action of COF-MOFs, the combination of HOFs with MOFs also showed superior biological and chemical activities to those of MOFs alone [39].

**Fig. 5:**
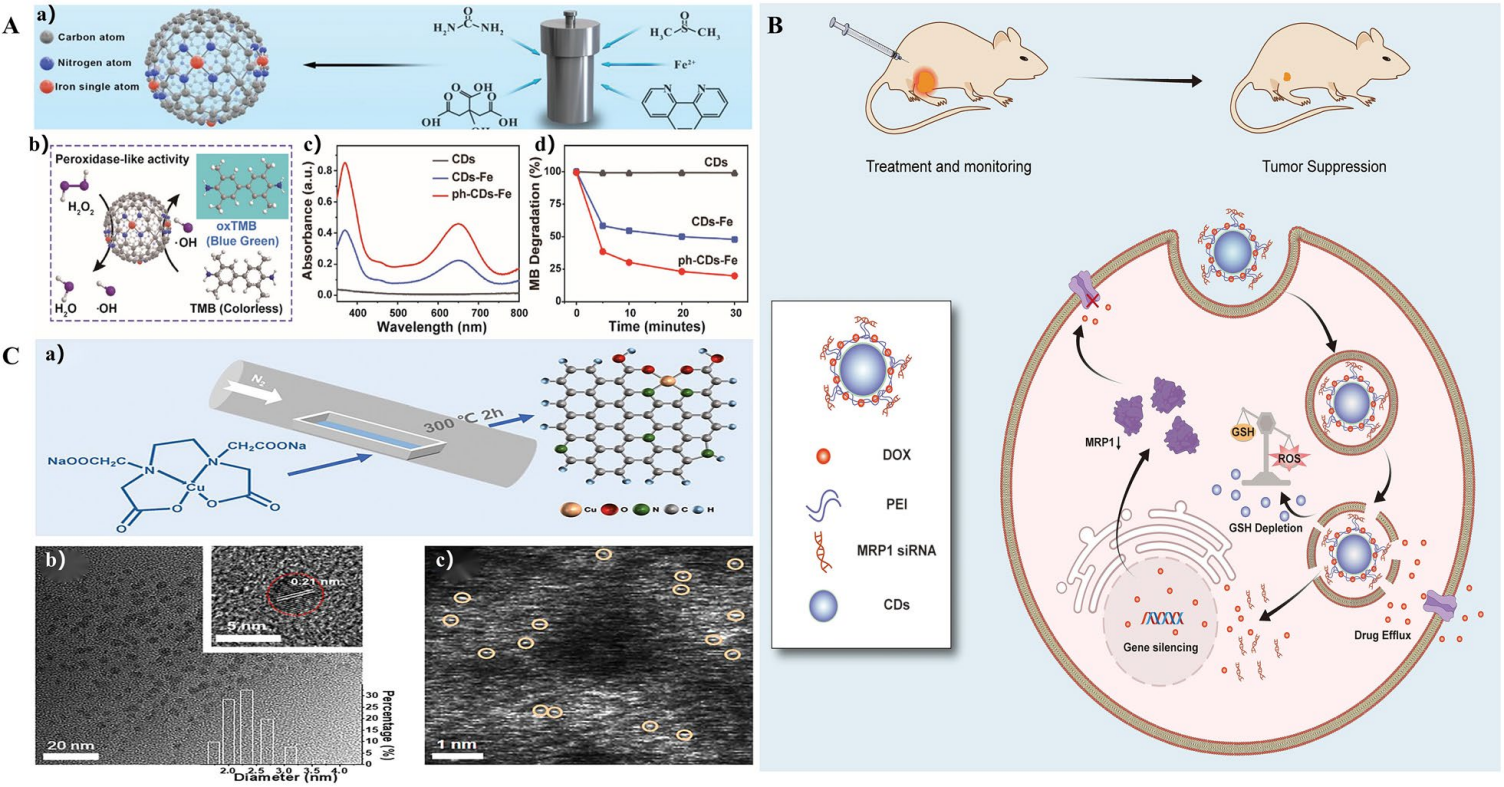
Optimization scheme for carbon dots structures.** A** Peroxidase-like activity of ph-CDs-Fe SAzyme. a) Schematic illustration of POD-like activity of CDs, Fe-CDs SAzyme, and ph-CDs-Fe SAzyme. b) POD-like activity of CDs, CDs-Fe, and ph-CDs-Fe SAzyme. c) Degradation of methylene blue (MB, 12.5 µg mL^−1^, pH 5.0) by CDs, Fe-CDs SAzyme, and ph-CDs-Fe SAzyme in the presence of H_2_O_2_ (5 mM). Reproduced with permission [45]. Copyright 2023. Wiley–VCH.** B** The schematic diagram delineates how CD-PEI-DOX-siMRP1 delivers doxorubicin to tumors and antagonizes chemoresistance by hindering drug efflux through knocking down MRP1 expression. Conversion of GSH to GSSG and subsequent ROS increase by CD-PEI oxidase and peroxidase activity further impairs MRP1 function. Collectively, CD-PEI-DOX-siMRP1 was capable of delivering drugs efficiently to tumor entities and retaining them in cells by hindering outflux of MRP1 through synergistic delivery of siRNA and perturbation of GSH-ROS balance. Reproduced with permission [46]. Copyright 2023. Dove Medical Press Ltd.** C** Synthesis and morphologies of Cu-CDs-300. a) Scheme of the calcining procedure for Cu-CDs-300 as an example. b) TEM and HRTEM images of Cu-CDs-300. c) Typical HAADF-STEM image of distributed single Cu atoms (orange circles) in Cu-CDs-300. Reproduced with permission [47]. Copyright 2023. Wiley–VCH

Carbon dots, a new type of carbon nanomaterial with luminescent properties, possess a rich source of raw materials, facile surface modification, low toxicit [40] and high biocompatibility [41]. Doping or loading individual metal atoms can boost the catalytic activity of carbon dots, including peroxidase activity, oxidase mimetic activity, and catalytic activity [10, 42]. To enhance the biosafety, controllability, and catalytic activity of single-atom nanozymes, carbon dots were introduced during the synthesis of SAzymes [43, 44]. By using a solvothermal technique, Han et al. [45] created carbon dots (CDs)-loaded single-atom iron nanozymes (ph-CDs-Fe SAzymes), which showed high POD activity (Fig. 5A). Yu et al. [46] used polyethyleneimine (PEI) to modify carbon dots, receiving better optical characteristics (Fig. 5B). Furthermore, by varying the reaction temperature, it was possible to optimize the various coordination structures of CD, thereby altering the bioactivity of the composites with SAzymes. For example, CD-loaded copper single-atom nanozymes with various coordination structures and peroxidase-like characteristics could be synthesized at various temperatures (Fig. 5C) [47]. The aforementioned research has demonstrated a connection between the carbon dot form and the catalytic site variation, which in turn influences the biological activity of the composites.

Because of their atomic characteristics and spatial arrangement, we found that materials that consist of crystalline frameworks, such as MOFs, COFs, and HOFs, are crucial for optimizing single-atom nanozyme complexes. MOFs structures possess a tendency to collapse in high-temperature environments, which is not conducive to a high degree of dispersion, and lead to changes in the oxidation state, phase purity and atomic level characterization of the metal, leaving the stability of the resulting nanozymes uncontrolled. Experimental data on COFs have collectively shown that COFs as a framework or carrier may cause complete or partial pore blockage on their surface, which significantly impacts the exposure of the active sites of the nanozymes. The inherent instability and structural recalcitrance are the primary obstacles to HOF development [48]. Previous research has conclusively demonstrated that the incorporation of functional groups typically shapes into new hydrogen bonds and alters the topology, which may have an effect on the function of SAzymes.

### Optimization of active center

The experimental findings reveal an unbreakable link between particular atomic metal centers and catalytic activity. Ou et al. [49] suspected that material properties are affected by the spatial location of monatomic atoms. To examine their photocatalytic antibacterial activity, they created Cu single-atom-site nanozymes both within and outside of polyheptazinimide nano-(PHI) platforms. The results show that the interlayer-localized copper material (CuL/PHI) has a broader antimicrobial effect on a wide range of bacterial strains compared to other spatially arranged materials, and can achieve the same antimicrobial effect as antibiotics. During the catalytic process, Rh(V)N_4_ and the preferentially formed Rh(V)-O-N_4_ structure can act as active centers, enabling the SAzyme to exhibit excellent POD-like activity. This structure improves catalyst utilization and significantly reduces reaction energy through the “two-sided oxygen chain” catalytic reaction pathway [50]. According to Wang et al. [51], metal-H_2_O_2_ interactions may have an impact on the catalytic activity of CAT. It was demonstrated that Ir-N_4_ had the strongest interaction with H_2_O_2_, the lowest H_2_O_2_ decomposition barrier, and the highest CAT-like activity out of the five active centers based on platinum group metals (M-N_4_, M = Ir, Ru, Rh, Pt, and Pd). In a manner similar to this, Cheng et al. [5] synthesized various active sites of metal-N to produce artificial metalloenzymes and a library of metalloenzyme imitators. Among the artificial metal nanozymes, they found that the Fe-like metal-centered nanozymes had the highest OXD-like activity, metal-centered nanozymes with a copper-like center demonstrated the highest level of POD-like activity.

Precious metals have strong catalytic activity and noble metal-precious metal bimetallic nanocatalysts have been reported. Fan et al. [53] wrapped a thin layer of palladium around gold nanorods (AuNRs) as the core to form Au@PdNRs, which showed notable oxidase-like activities under dark environment and plasma resonance excitation, and the enhanced catalytic activity was mainly due to the full exposure of the Pd surface used for catalysis. Both non-precious and precious characteristics of metals are displayed by nanozymes made of both types of metals. An ultrasmall single-atom Pt/CeO_2_ with continuous catalytic activity was created by Yan et al. [54]. The highly ordered arrangement of metal atoms in the resultant nanocage allowed the SAzymes to have POD- and GSH peroxidase like (GSH-Px-like) catalytic activity and efficient ROS production under ultrasonic irradiation. The single-atom Pt generated a selective distribution on the crystalline surface of CeO_2_. Similarly, Zhong et al. [55] demonstrated that PtCu_3_ nanocages can also be used as copper-based nanomaterials as novel acoustic sensitizers to generate ROS under irradiation.

It can be inferred from several experiments that the synergistic action of metals in multimetallic catalysts may result in greater selectivity and catalytic efficiency than in monometallic catalysts. A Cu/Zn bimetallic atom nanoconjugate enzyme (Cu/PMCS) was created by Liu et al. [56]. The findings indicated that doping Cu atoms increased the catalytic activity and GSH depletion of this nanoconjugate enzyme, which improved its anti-tumor capacity. Additionally, according to Lv et al. [57], the peroxidase catalytic activity of nanorods containing Au and Pt bimetallic atoms was significantly higher than that of nanozymes containing single metal atoms. Therefore, it is clear that adding more metal sites will increase the catalytic activity of nanozymes. The catalytic activity of single-atom nano-enzymes is connected with the ratio of metal atoms in addition to the influence of the quantity of metal atoms. Cai et al. [58] discovered that the shape, structure, and content of the products are influenced by the Au–Pd synthesis atomic ratio. In model oxidation reactions, 0D/2D Au–Pd nanocomposites demonstrated significantly increased peroxidase-mimetic catalysis. This was theorized to be directly related to changes in the electronic structure of Au–Pd and an increase in its specific surface area ratio.

Overall, a number of experiments have been carried out recently with the aim of enhancing the enzyme-like catalytic activity of single-atom nanozymes. These experiments range from the different types of single atoms and their spatial positions corresponding to different kinds of enzyme-like activities to the interactions between polyatoms and the changes in the ratio of polymetallic atoms significantly affecting the catalytic activity, which have produced encouraging results, and we anticipate the emergence of more effective and straightforward optimization based on the results.

### Synthesis strategy of SAzymes

#### Synthesis process

**Fig. 6:**
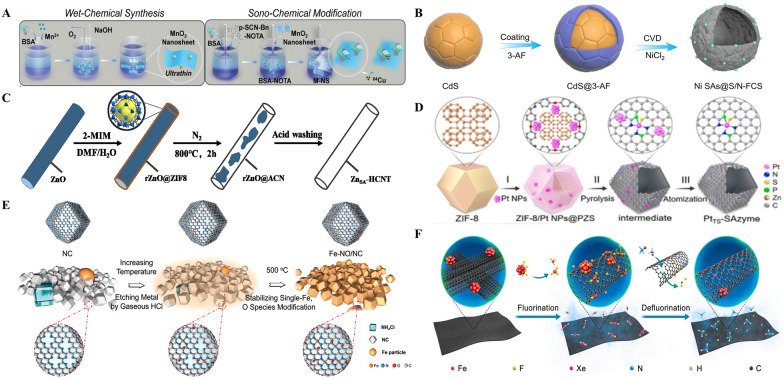
Strategies for the synthesis of single-atom nanozymes.** A** Schematic illustration of two-step synthesis of M-NS. First, a novel “wet-chemical method” was applied to form 2D nanosheets under the direction of BSA. Second, a unique “sono-chemical method” was introduced to further enhance the stability, biocompatibility, and surface functionality of the M-NS. Reproduced with permission [61]. Copyright 2019. Wiley–VCH.** B** Schematic of the synthetic process for the Ni SAs@S/N-FCS. Reproduced with permission [65]. Copyright 2022. Wiley–VCH. **C** Schematic diagram of the synthesis process of ZnSA-HCNT. Reproduced with permission [66]. Copyright 2022. Wiley–VCH.** D** Illustration of the preparation process of PtTS-SAzyme. Reproduced with permission [67]. Copyright 2021. American Chemical Society.** E** Schematic of the Preparation Strategy for Fe–NO/NC. Reproduced with permission [68]. Copyright 2020. American Chemical Society.** F** Schematic showing the preparation of the SAFe-SWCNT film. Reproduced with permission [69]. Copyright 2021. Elsevier

SAzymes have been synthesized in various ways, such as by constructing defect sites on metal hydroxide/oxide substrates, by creating spatially restricted domain effects on the substrate material, etc., which basically improve the interaction of metal atoms with the coordinating heteroatoms. For SAzymes, the most classical synthesis strategy is wet chemistry, including coprecipitation, impregnation, and ion exchange [59]. Using zeolite Y as a carrier, Cheng et al. [60] developed a straightforward wet impregnation technique to produce a stable nanocomposite (CeO_2_/Y). Tang et al. [61] developed a novel one-step wet chemical process after optimizing a number of investigations, and they used BSA to guide the formation of two-dimensional nanosheets (Fig. 6A). To further improve the stability, biocompatibility, and surface functioning of the resulting BSA-NOTA-modified MnO_2_ nanosheets (M-NS), they also presented an acoustic chemical synthesis method.

SAzymes can be prepared by direct pyrolysis for those precursor metal blocks or metal materials composed of thermodynamically unstable metals. Most types of SAzymes in the current study, especially those based on nitrogen-doped carbon materials, are mainly prepared by pyrolysis [62]. In the direct pyrolysis pathway, it is of utmost importance to break the metal–metal bonds of the precursor carriers and to minimize the escape of metal atoms at high temperatures [63]. Huang et al. [64] prepared single Fe atom nano-enzymes with Fe-N_5_ active sites by pyrolyzing MOFs at 900 °C under N_2_ atmosphere. Zhao et al. [65] pyrolyzed at 950 °C to generate Ni single-atom catalysts (Fig. 6B). Similar to this, Li et al. [66] created zinc monoatomic nanozymes through the pyrolysis and high-temperature adsorption reactions of zinc with imidazole (Fig. 6C). Coincidentally, Chen et al. [67] reversed the thermal sintering process and directly atomized platinum nanoparticles (Pt NPs) into single atoms to produce high-performance nanozymes (Fig. 6D). However, the direct pyrolysis of MOFs has its drawbacks: during the pyrolysis process, the structure of MOFs tends to collapse due to high temperatures, leading to volume contraction, which is unfavorable for the high dispersion of monatomic metals.

In order to encourage the in-situ formation of volatile metal species in metal nanoparticles and induce their anchoring to the carrier, the gas migration technique entails the introduction of corrosive or reducing gases, such as ammonia, phosphine, and hydrogen chloride. Using a combination of NC, Fe NPs, and NH_4_Cl as precursors for pyrolysis, Li et al. [68] found that the resulting FeCl_2_ could be readily vapor-diffused into the carriers at 500 °C, forming Fe monoatoms (Fig. 6E). In a similar manner, Yu et al. [69] produced SAFe-SWCNT thin films by loading Fe NPs into single-walled carbon nanotubes (SWCNT) and then annealing the SWCNT during the process of XeF_2_ introduction **(**Fig. 6F**)**.

#### Methods for dispersing metal atoms

Single-atom metalloids can be obtained directly from precursors, while achieving a uniform dispersion of individual atoms and stopping them from migrating and aggregating to form NPs are the main goals of the synthesis of SAzymes [70]. UP to date, approaches based on enhanced metal-carrier interactions to capture isolated metal atoms on carriers mainly include spatial confinement, defect anchoring strategy and coordination stabilization. In addition, the research frequently uses chemical etching, gas migration, direct pyrolysis, and the electrochemical deposition approach.

##### Spatial confinement

**Fig. 7:**
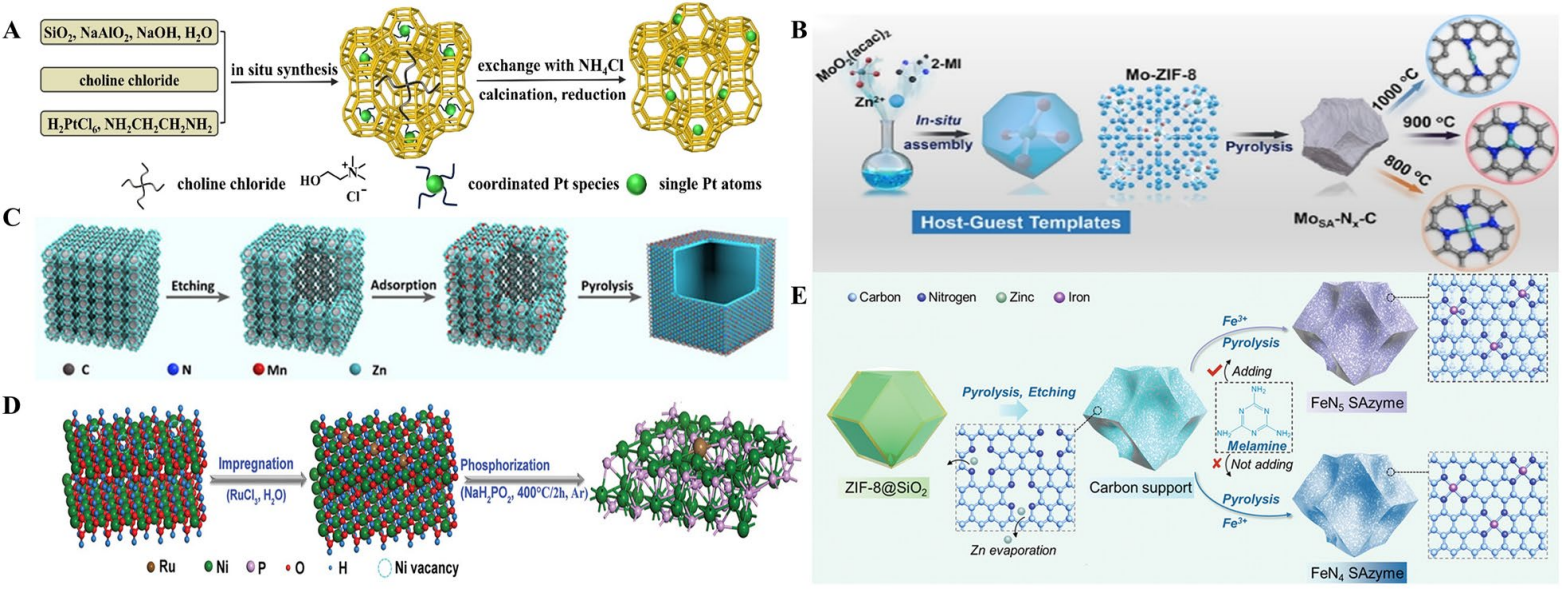
Methods for dispersing metal atoms: spatial confinement, defect anchoring strategy and coordination stabilization.** A** Illustration of the synthesis strategy to selectively encage single Pt atoms into the six-membered rings of SOD cages within Y zeolite. Reproduced with permission [71]. Copyright 2022. Wiley–VCH.** B** Schematic illustration for the fabrication strategy of MoSA-Nx-C catalysts. Reproduced with permission [72]. Copyright 2021. Elsevier.** C** Coordination of monoatomic manganese and nitrogen atoms in a hollow zeolitic imidazolite skeleton to construct Mn/PSAE. Reproduced with permission [73]. Copyright 2021. Wiley–VCH.** D** Schematic diagram for the synthesis of Ni5P_4_-Ru. Reproduced with permission [74]. Copyright 2020. Wiley–VCH.** E** Schematic illustration of synthesis process for FeN_5_ SAzyme. Reproduced with permission [75]. Copyright 2022. Wiley–VCH

The essence of spatial confinement lies in the encapsulation and separation of the metal precursor to immobilize the active center of the metal atom. Individual Pt atoms were carefully encapsulated in the six-membered ring of a Sodalite (SOD) cage within a Y molecular sieve by Chen et al. [71] using a template-guided approach. (Fig. 7A). In order to create single-atom Mo SAzymes with varying coordination numbers, Wang et al. [72] enclosed MoO_2_(acac)_2_ molecules in ZIF-8 pores. Following pyrolysis, the Mn^2+^ absorbed by the hollow structure was transformed into single-atom Mn sites in the SAzymes. The molybdenum single-atom nanosize enzyme (MoSA-Nx-C) was established and verified for its peroxidase-like specificity (Fig. 7B). By coordinating monoatomic manganese and nitrogen atoms in a hollow zeolite imidazolite skeleton, Yang et al. [73] created a polyethylene glycolated manganese-based SAE (Mn/PSAE). Mn/PSAE demonstrated notable therapeutic effects by producing a range of ROS and photothermal activities through stimulation of the tumor microenvironment (Fig. 7C).

##### Defect anchoring strategy

Immobilizing metal atoms can also be accomplished effectively by carrier defects. By using crystal surface defects of CeO_2_ clusters to trap Pt atoms, Yan et al. [54] successfully synthesized single-atom Pt/CeO_2_ clusters for the treatment of traumatic brain damage. He et al. [74] constructed Ni(OH)_2_-rich nickel vacancies with RuCl_3_ and obtained the target Ni_5_P_4_-Ru SAC by subsequent phosphorylation treatment, where strong interactions between the nickel vacancy defects and Ru cations enhanced the Ru doping rate (Fig. 7D). Defects on the carbon carriers that served as anchor sites for the insertion of Fe atoms were created by Xu and associates [75]. The Fe atoms inserted into the flaws were immobilized by the N species in the NC substrate, and the FeN_5_ SAzymes exhibited superior peroxidase-like activity in the tumor microenvironment. ZIF-8 precursors covered with SiO_2_ that were pyrolyzed to cause Zn volatilization (Fig. 7E).

##### Coordination stabilization

With regard to the ligand stabilization doctrine, it has been shown that metal-nonmetal bonds can significantly enhance metal-carrier interactions [70]. In order to improve the metal-carrier interactions and the charge redistribution of the surrounding atoms, Au atoms were added to NiFe layered double hydroxides by Zhang et al. [76], where Au atoms on O atoms produced the hydroxides. As a result, catalytic performance was enhanced. This led to improved catalytic performance. Ag atoms were uniformly distributed on TiO_2_ carriers by Wang et al. [77], who then created Ag SAzymes with potent antiviral properties by means of strong Ag–O metal-carrier interactions. In order to facilitate the effective oxidative breakdown of propranolol in water, single Fe atoms were trapped in two-dimensional MoS nanosheets (Fe MoS) by Huang et al. [78] as extremely reactive catalysts for the non-homogeneous activation of sulfites.

The spatial confinement approach encapsulates fleeing metal atoms primarily through metal-carrier interactions. The stability of SAzymes is mostly caused by the interaction between the metal and the carrier [79]. However, the matrix framework in the wet-chemical synthesis method hides some of the metal active centers, resulting in a reduced usage of monoatomic metals. Similar to how the gas migration strategy chooses certain gases with corrosive or reducing properties to make the metal nanoparticles more volatile and increase their anchoring efficiency, the direct pyrolysis strategy uses high temperature to forcibly break strong metal–metal (M-M) bonds in the metal nanoparticles in the metal nanozymes.

## Bioenzymatic-like catalytic activity of SAzymes

**Fig. 8:**
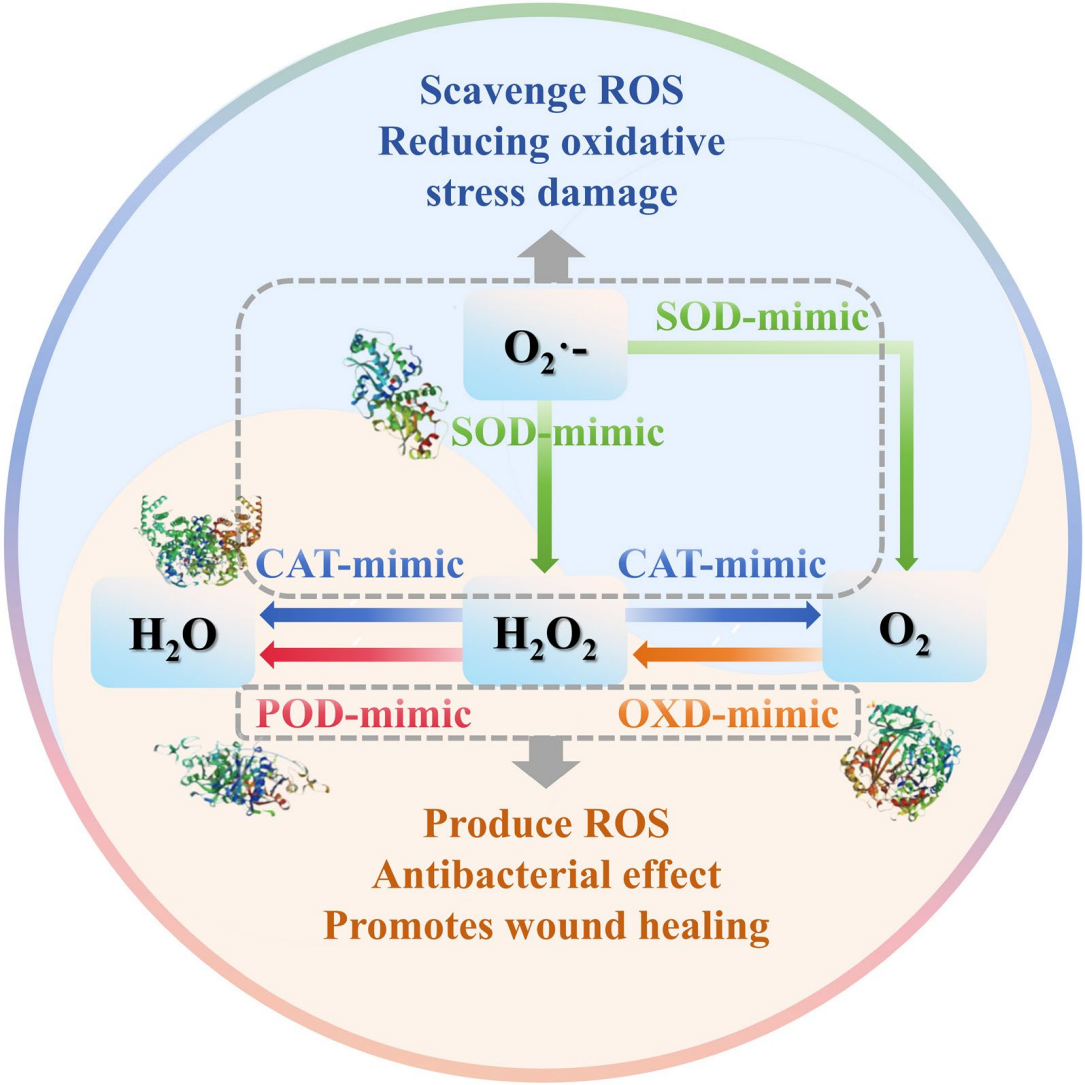
Schematic diagrams of the main enzyme-like catalytic activities of various nanozymes. POD-like activity of the nanozymes catalyzes peroxidation reaction using H_2_O_2_ as substrate. OXD-like activity nanozymes catalyze the generation of H_2_O_2_ in the presence of O_2_. POD-like activity and OXD-like activity nanozymes usually promote the production of ROS in organisms, resulting in a bactericidal effect. CAT-like activity helps to catalyze the decomposition of H_2_O_2_ into O_2_ and H_2_O. SOD-like activity mainly catalyzes the disproportionation of superoxide anion radicals (O2·-) into H_2_O2 and O_2_.POD-like activity and OXD-like activity nanozymes mainly scavenge excess ROS in the body and mitigate oxidative stress damage. *OXD* oxidase, *POD* peroxidase, *CAT* catalase, *SOD* superoxide dismutase, *ROS* reactive oxygen species

Artificial enzymes of different types and structures have different catalytic mechanisms, which lead to different final catalytic results (Fig. 8). This section will discuss several oxidoreductases mimicked by SAzymes that have been identified in recent studies, including POD, OXD, CAT, SOD, and GSH-Px (Table 1).

**Table 1:** Summary of various bioenzymatic-like catalytic activity of SAzymes

Mimic function	Materials	Active site	Applications	Refs
POD	Fe-N-C SAzymes	Fe	Bio-detection	[81]
	Fe-N-C	Fe	Bio-detection	[82]
	CNT/FeNC	Fe	Bio-detection	[83]
	Cu-N-C	Cu	Bio-detection	[84]
	MoSA-Nx-C	Mo	Bio-detection	[72]
	Zn- ZIF-8	Zn	Anti-bacterial effect	[85]
OXD	Fe-N-C	Fe	Bio-detection	[86]
	Fe-N-C SAzymes	Fe	Bio-detection	[87]
	Ru SAzymes	Ru	Anti-tumor therapy	[88]
	Mn/PSAE	Mn	Anti-tumor therapy	[73]
	FeN_5_SA/CNF	Fe	Anti-bacterial effect	[64]
CAT	Co/PMCS	Co	Anti-bacterial effect	[89]
	OxgeMCC-r	Mn, Co	Anti-tumor therapy	[28]
	SAFe-NMCNs	Fe	Anti-tumor therapy	[90]
	Fe-SAs/NC	Fe	Anti-inflammatory effect	[91]
SOD	Pt/CeO	Pt, Ce	Anti-inflammatory effect	[54]
	Co/PMCS	Co	Anti-bacterial effect	[89]
	Au24Cd1	Au, Cd	Anti-inflammatory effect	[92]
	Cu-HNCS	Cu	Anti-tumor therapy	[93]
GSH-Px	Cu SASs/NPC	Cu	Anti-tumor therapy	[94]
	Cu/ N-doped carbon	Cu	Anti-tumor therapy	[95]
	Fe/ N-doped carbon	Fe	Anti-tumor therapy	[96]

### POD-mimetic activity

With H_2_O_2_ serving as the electron acceptor, the POD-like activity of SAzymes catalyzes the synthesis of -OH, which severely damages cancer cells through oxidative stress by producing copious amounts of reactive oxygen species. POD-like SAzymes are widely distributed, and their nanomaterials contain a wide variety of transition metals, primarily Fe, Cu, Au, Pt, Co, and Ce [80]. Research has demonstrated that Fe-N-C single-atom nano-enzymes (Fe-N-C SAzymes), which are prepared by high-temperature calcination, have peroxidase-like activity [81]. More precisely, their enzymatic activity is less than that of peroxidase-like activity and is more akin to that of natural metalloproteinases. Fe-N-C SAzymes can detect H_2_O_2_ colorimetrically and have a better catalytic efficiency, which makes them more useful in biology. For example, Fe-N-C single atom nanozymes have remarkable peroxidase mimic activity. Butyrylcholinesterase (BChE) activity can be highly sensibly biosensitized with these SAzymes. POD activity is also shown by other SAzymes that include Fe single atoms as their active centers [82]. New SAzymes based on carbon nanotubes loaded with Fe single atoms (CNT/FeNC) were used to detect H_2_O_2_, ascorbic acid, and glucose. These enzymes also showed good peroxidase-like activity [83]. According to the previously cited Wu et al. [84], Cu-N-C was produced on carbon nanosheets with a high concentration of Cu sites by replacing Fe in SAzymes with Cu, and it was shown that the peroxidase activity remained present. Furthermore, due of the dense dispersion of active copper atoms (≈5.1 wt%), the Cu-N-C SAzymes showed remarkable activity in mimicking natural peroxidase. Excellent POD activity was also shown by a series of single-atom nano-enzymatic liquids with different metal active centers. Furthermore, research on molybdenum single-atom nanozymes (MoSA-Nx-C) showed that the quantity of ligands at a particular molybdenum site controlled the specificity of the enzyme similar to peroxidase, offering a useful approach for the logical construction of the targeted nanozymes [72]. Carbon nanomaterials derived from zinc-based zeolite imidazolate frameworks (ZIF-8), which have zinc atoms dispersed atomically, can be used to make efficient single-atom peroxidase mimics [85].

### OXD-mimetic activity

Using O_2_ as the reaction substrate, OXD-like activities are a class of redox reactions that can imitate amino acid oxidase (AAO), uric acid oxidase (UOX), and glucose oxidase (GOX), depending on the kind of hydrogen donor [80]. Whereas SAzymes with limited reducing activity convert O_2_ to H_2_O, those with significant reducing characteristics convert O_2_ straight to H_2_O_2_. To examine the structure–activity link, Xu et al. [86] looked at Fe-N-C single-atom catalysts with two distinct coordination structures (NG-Heme and G-Heme). In particular, the contact between the active site and the intermediate can eventually boost its intrinsic oxidase activity. It was discovered that NG-Heme exhibits stronger oxidase activity than G-Heme. It has a high sensitivity for colorimetric carcinoembryonic antigen detection in clinical settings. Other research had looked into the performance of Fe-N-C SAzymes as oxidase-like SAzymes. These enzymes have atomically dispersed metal active sites that can be activated to produce reactive oxygen species [87]. Ru single atom loading based on carbon dots has produced SAzymes with strong stability, outstanding biocompatibility, and great activity [88]. Ru SAzymes can function as surrogates for glutathione oxidases, peroxidases, and other oxidative enzymes. ROS formation and glutathione depletion can be catalyzed simultaneously by them, which can exacerbate ROS-induced cell damage and ultimately lead to cancer cell death—a crucial stage in tumor treatment. Zhu et al. [73] reported that a manganese-based PEGylated SAN (Mn/PSAE) could efficiently produce large amounts of harmful ROS and mimic the different catalytic capabilities of OXD, POD, and CAT enzymes in addition to having better photothermal properties. FeN_5_, when contained within a carbon nanoframework (FeN_5_SA/CNF), possesses a well-defined active part and important synergistic effects among its single-atom nano-enzymatic active centers, which confer outstanding oxidase-like activity and multifunctional antimicrobial activities [64]. This implies that the axial N coordination in FeN_5_SA/CNF has an oxidase-like driving effect, increasing its catalytic activity relative to other SAzymes.

### CAT-mimetic activity

An important antioxidant enzyme in the metabolism of ROS and H_2_O_2_ is CAT. Cancer cells suffer significant oxidative damage when there is an abundance of ROS. By breaking down endogenous H_2_O_2_ into O2, the CAT-like activity of SAzymes helps to improve the hypoxic environment around tumors in cancer cells. CAT-like nanozymes mimic natural CAT by generating H_2_O and O_2_ from H_2_O_2_ as substrates, providing electrons from the catalytic activity center to align and disproportionate H_2_O_2_. With atomically dispersed active centers, Cao et al. [89] constructed Co/PMCS as an enzyme-mimicking single-atom catalyst. It atomically dispersed active centers, which can eliminate H_2_O_2_ by mimicking superoxide dismutase, catalase, and glutathione peroxidase, and its elimination efficiency is significantly higher than that of other nanozymes. With single-atom ruthenium serving as an active catalytic site anchored in the metal–organic framework Mn_3_[Co(CN)_6_]_2_, the self-assembled photodynamic therapy nanoreagent OxgeMCC-r SAzymes produces OxgeMCC-r with a high loading capacity and CAT-like activity. Endogenous H_2_O_2_ reacts with single-atom Ru in OxgeMCC-r to generate O_2_ in situ, reducing hypoxic conditions in the tumor microenvironment [28]. The dual catalytic activity of single-atom iron-dispersed N-doped mesoporous carbon nanospheres (SAFe-NMCNs) nanozymes imitate both oxidase and peroxidase [90]. Experiments conducted in vitro and in vivo shown that the SAFe-NMCNs nanozymes could effectively limit the proliferation of tumor cells and had a synergistic therapeutic effect when combined with photothermal-enhanced nanocatalytic therapy. It is possible to mimic the actions of catalase (CAT) and SOD by anchoring atomically dispersed Fe-N_4_ sites on N-doped porous carbon materials (Fe-SAs/NC) [91]. Because of this, Fe-SAs/NC can work as a dual-purpose single-atom-based enzyme (SAzyme) that scavenges reactive oxygen species (ROS) and gets rid of extra ROS that are created when cells are under oxidative stress.

### SOD-mimetic activity

The transition metal elements Cu, Zn, Mn, and Fe are the key components of antioxidant metalloenzymes, which are found in organisms and regulate ROS levels in cells. These enzymes have SOD-like activity. Consequently, Cu, Mn, and Au are also the primary components of SOD-like activity in nanozymes including transition metal elements [80]. Because of their special qualities, carbon-based SOD mimetic nanozymes have lately been employed as promising antioxidant nanotherapeutics. The majority of reactive oxygen species (ROS) in the body are produced by superoxide anions, which can be scavenged by SAzymes having SOD-like activity. Yan et al. [54] developed a single-atom Pt/CeO with long-lasting catalytic activity, and the single-atom Pt greatly increased the endogenous CAT activity, resulting in greatly increased scavenging activity and enzyme-like activity of RONS of the final SAzymes compared to other materials. Co/PMCS was found to have atomically dispersed ligand-unsaturated active centers and hence also possesses SOD-like, CAT- and glutathione peroxidase activities [89]. In the end, this lowers pro-inflammatory cytokine levels and shields organs from harm. Due to its wealth of electronic energy levels and surplus of transition metal electronic states, Au offers a strong foundation for the development of atomic-level enzymes. It was demonstrated that the activities of Au_25_, Au_24_Cu_1_, and Au_24_Cd_1_ are similar to those of GSH-Px, CAT, and SOD, respectively [92]. Au_24_Cu_1_ reduces peroxides in the damaging brain, whereas Au_24_Cd_1_ prioritizes the use of superoxides and significantly reduces inflammatory factors. In order to enhance the suppression of tumor growth, Lu et al. [93] created an N-doped carbon sphere doped with monoatomic copper, called Cu-HNCS, whose SAzymes may directly catalyze the breakdown of oxygen and hydrogen peroxide into ROS. And more research revealed that monoatomic copper was the primary source of the strong catalytic activity of Cu-HNCS.

### GSH-Px-mimetic activity

The primary mechanism of catalysis in single-atom nanozymes exhibiting glutathione peroxidase activity involves the depletion of intracellular glutathione superoxide (GSH), resulting in the elimination of microbes and cancerous cells. Cu single-atom sites/N-doped porous carbon (Cu SASs/NPC) can function as GSH-Px-like nanozymes, which can deplete GSH in bacteria and hence greatly enhance the bactericidal effect. Cu SASs/NPC have a greater glutathione (GSH) depletion capacity than non-Cu-doped NPC [94]. In addition, copper SAzymes based on a bionic single-atom nano-enzyme system have been prepared in other studies and validated to have excellent POD-like activity [95]. It demonstrated effective tumor targeting both in vivo and in vitro, preventing the ability of cells to synthesize GSH from the source. An adaptive iron mutation platform developed by Cao et al. [96] builds upon single-atom nanozymes (SAzymes). By enabling SAzymes to deplete GSH in tumor cells on demand, the platform speeds up safe and selective iron apoptosis. Studies on breast and colon cancers have shown evidence of this adaptive anti-tumor response.

## Biological applications of SAzymes in gastrointestinal (GI) diseases

**Fig. 9:**
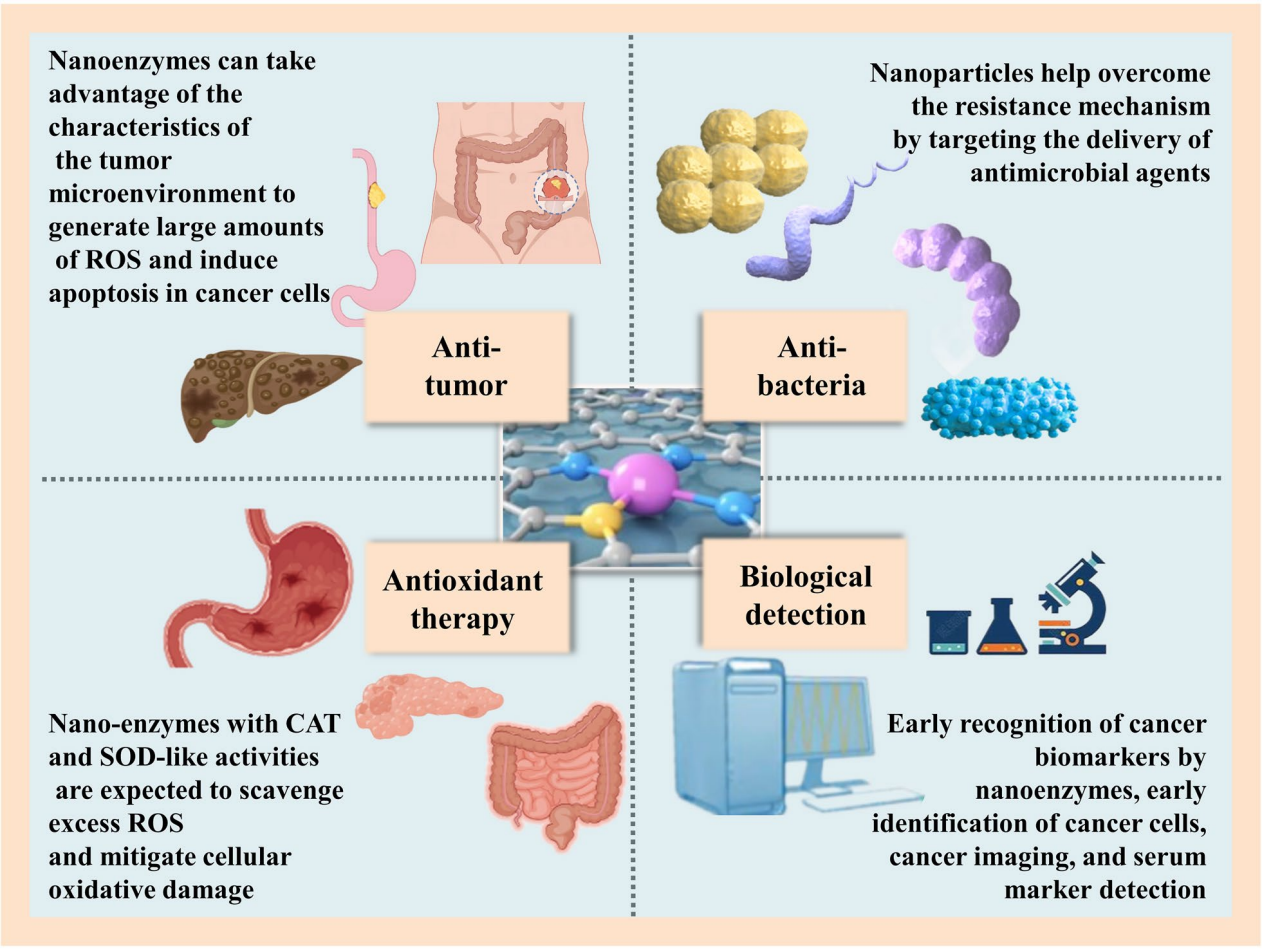
Various biological applications of nanozymes in gastrointestinal diseases

While nanozymes have catalytic activity similar to that of genuine enzymes, they suffer from a number of serious problems, such as insufficient substrate selectivity, confusing architectures, and imprecise catalytic processes [97]. SAzymes have the advantages of facile separation, homogenous active sites, customizable coordination environments, and high atom utilization because they allow for the rational design of the coordination environment and the selection of metal atoms and their valence states. In particular, nitrogen-doped carbon-loaded SAzymes have structurally similar metal-Nx(M-Nx) sites to those of natural enzymes, and thus are considered to have a wide range of prospects for mimicking natural enzyme activities [85]. This section summarizes the biological application areas of nanozymes, primarily based on recent basic research, as illustrated in the image. These fields include bioassays, antibacterial, anticancer therapy, and anti-inflammatory effects (Fig. 9).

### Antibacterial application

ROS and reactive oxygen species (RNS) are the most common free radicals, and excessive accumulation of free radicals can help nanoparticles to fight against microorganisms or to reduce microbial resistance [98]. SAzymes can undergo a Fenton-like reaction, which is utilized to kill bacteria by the ROS produced by the reaction, and have demonstrated excellent bactericidal effects in GI diseases (Table 2).

**Table 2:** Biological applications of SAzymes in Gastrointestinal (GI) Diseases

Materials	Enzyme-like activities	Active site	Applications	Refs
PMCS	POD	Zn	Antibacterial	[100]
Cu SASs/NPC	POD	Cu	Antibacterial	[56]
SAF NCs	POD	Fe	Antibacterial	[101]
FeSAs@Sa.M	POD, OXD	Fe	Antibacterial	[102]
l-Arg@Cu-SAzymes	CAT, OXD	Cu	Antibacterial	[103]
FeN_5_SA/CNF	OXD	Fe	Antibacterial	[64]
Co/PMCS	SOD, CAT, GSH-Px	Co	Antibacterial	[89]
BSA-Cu SAzymes	GSH-Px, Fenton-like	Cu	Antitumor	[105]
PFeSA@AS	Hemoglobin mimic	Fe	Antitumor	[106]
Cu-PBMCs	CAT	Cu	Antitumor r	[107]
Ir-N_5_ SA	POD, OXD, CAT, NOX	Ir	Antitumor	[108]
Fe@Fe_3_O_4_	POD, CAT	Fe	Antitumor	[109]
Au-Fe SAzymes	GOD	Au, Fe	Antitumor	[111]
Pd SAzyme	POD, GSH-Px	Pd	Antitumor	[112]
IrN_4_-S-TMN_4_ (TM = Co, Rh, Pd)	CODH	Ir, Co/ Rh/Pd	Antitumor	[110]
macDNA-Fe/PMCS	POD, OXD	Fe	Antitumor	[96]
Fe-N/C SACs	POD, OXD, GSH-Px, CAT	Fe	Antioxidative	[114]
Co-SAzymes	SOD, CAT, GSH-Px	Co	Antioxidative	[89]
Cu SAs/CN	APX	Cu	Antioxidative	[115]
Pt@PCN_222_-Mn	SOD, CAT	Pt	Antioxidative	[116]
pFe SAzymes-GSH	OXD	Fe	Biological Detection	[119]
Apt/Fe Ne C SAzymes	POD	Fe	Biological Detection	[120]
A-Co-NG SAzymes	OXD	Co	Biological Detection	[121]
Fe-SAzyme	POD	Fe	Biological Detection	[122]
Co SAzyme	Fenton-like	Co	Biological Detection	[123]

Due to their broad-spectrum and drug-free antibacterial capabilities, single-atom nanozymes with antimicrobial activity have been increasingly significant in antimicrobial therapy in recent years [99]. *Escherichia coli*, *Staphylococcus aureus*, and some multidrug-resistant bacteria (MDR) are also common pathogens in GI disorders. The zinc-based ZIF-8 with atomically dispersed zinc atoms was reported by Xu et al. [100] and its derived carbon nanomaterials can be used as effective single-atom peroxidase mimics. Lastly, it was confirmed that SAzyme with unsaturated Zn-N_4_ sites is a powerful antimicrobial agent for wound treatment. It has strong antibacterial action against Pseudomonas aeruginosa as well as outstanding peroxidase-like activity. They conducted an analysis based on the particular mechanism by which Ag single atoms with high electrical conductivity could stimulate the MnO_2_ oxygen vacancies, hence facilitating the entry of reactive compounds that are photocatalytic. In addition, the photothermal conversion efficiency is enhanced by the catalysis of single-atom Ag, leading to the enhancement of the redox properties of the crystalline materials. Additionally, it has been investigated that Cu single-atom site/N-doped porous carbon (Cu SASs/NPC) was discovered to exhibit POD-like activity after being effectively synthesized using a sequence of pyrolysis-adsorption procedures [56]. The POD activity was greatly increased by doping the material with a single atom of Cu. Additionally, photo-thermal characteristics of the materials were specifically and simultaneously optimized to speed up the consumption of GSH. This synergistic effect enabled Cu SASs/NPC to exhibit excellent antimicrobial properties against *Escherichia coli.* and *methicillin-resistant S. aureus* (MRSA). The effective in vitro antibacterial and in vivo anti-infective qualities of single iron atom nanocatalysts have also been emphasized in a number of research conducted in recent years. Huo et al. [101] created nitrogen-doped amorphous carbon (SAF NCs) nanocatalysts attached with single iron atoms to cause peroxidase-like activity in the presence of H_2_O_2_, which efficiently eliminated Staphylococcus aureus and *Escherichia coli*. In order to eradicate intracellular MRSA, Liu et al. [102] created the highly sought-after missile-like nanotherapeutic medication FeSAs@Sa.M. and produced extremely toxic ROS through enzymatic activity at the center of FeSAs. A multifunctional Cu single-atom nanozyme (l-Arg@Cu-SAzymes) loaded with l-arginine was created by Qiu et al. [103]. This strategy was thought to be a promising way to treat MDR infection because it synergized with ROS and RNS to give the therapeutic system a strong antimicrobial efficacy and an enhanced tissue remodeling ability. In addition, antimicrobial activities against *Escherichia coli* [64]*.* and MRSA [89] have been demonstrated in ongoing experiments.

Despite the fact that ROS typically cause great harm to bacterial cells by damaging their cell membranes, as demonstrated by the aforementioned research, single-atom nanozymes are crucial for antibacterial applications primarily because of their biomimetic properties (particularly their POD-like activity).

### Antitumor therapy

One of the primary mechanisms for inducing apoptosis in tumor cells is the generation of ROS. In the biological arena, particularly in the area of anti-tumor therapy, they have achieved remarkable strides due to the multiple mimetic enzyme activities of SAzymes and the simultaneous creation of multiple ROS [104].

**Fig. 10:**
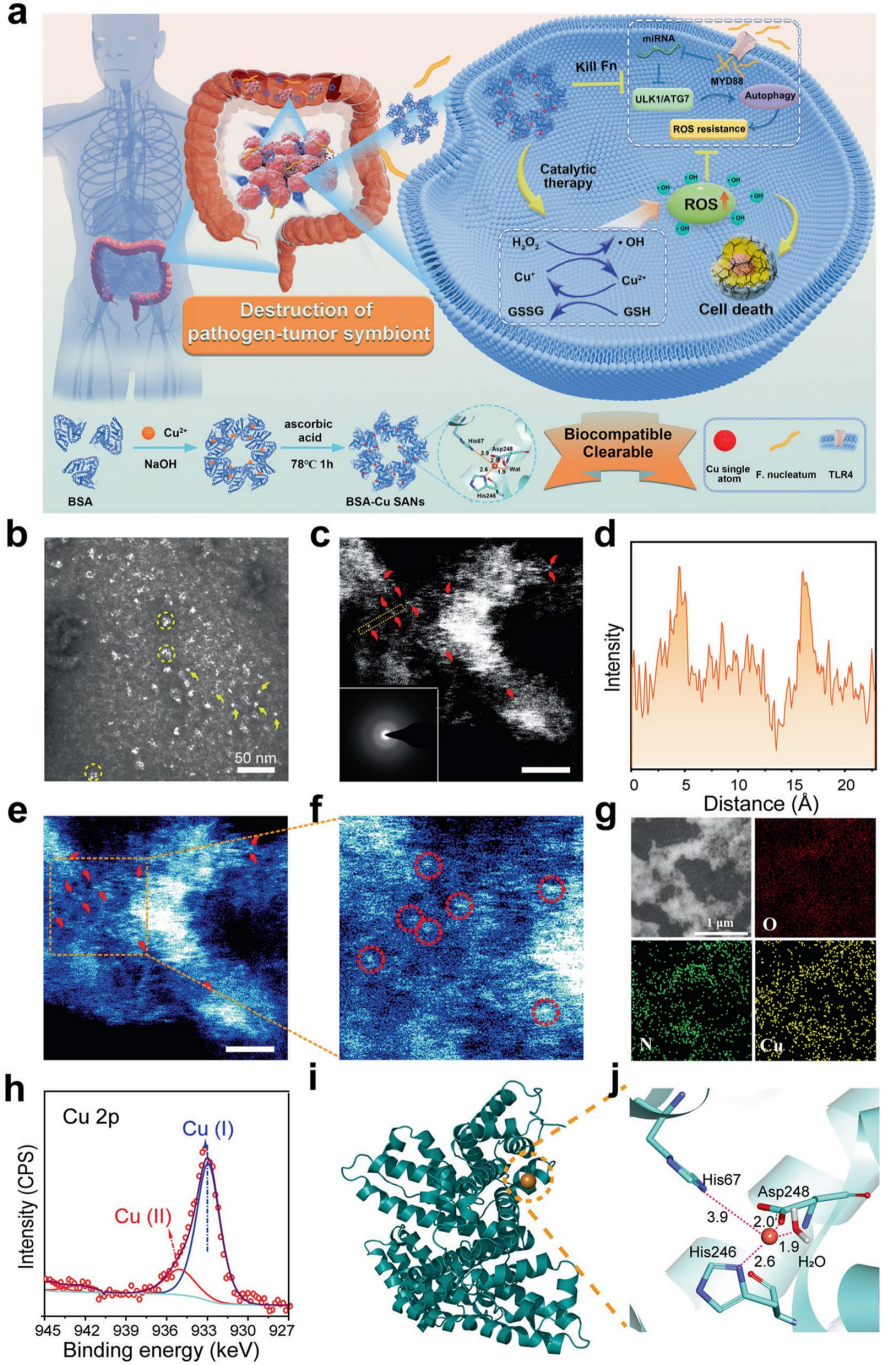
Formation and characterization of BSA-Cu SAN. **a** Schematic illustration of the synthesis of BSA-Cu SAN and its function of destroying pathogen-tumor symbionts for antitumor therapy, **b** Negative-stain electron microscopy image of BSA-Cu SAN. White bots represent BSA-Cu SAN, some of which are marked by yellow arrows. **c** Atomic HAADF image of BSA-Cu SAN with SAED pattern inset in the bottom left corner and partial single Cu atoms highlighted in yellow dash circles. **d** The intensity spectrum image along the distance in the yellow dash tangle in (**c**). **e** Pseudo-color image of corresponding intensity of (**c**). **f** The enlarged image of (**e**). **g** Elemental mapping of BSA-Cu SAN. **h** Cu 2p XPS spectra of BSA-Cu SAN. **i** Binding site of Cu^+^ on BSA based on the highest docking score. **j** Protein-ligand interaction diagram for Cu^+^. Reproduced with permission [105]. Copyright 2023, Springer Nature

Wang et al. [105] designed a protein-supported single-atom copper nanozyme (BSA-Cu SAzymes), which possesses ROS-generating and GSH-depleting effects, to effectively restore the elevated autophagy level of F. nucleatum and the ROS resistance of the tumor cells in situ to synergistically killing colorectal cancer (CRC) cells (Fig. 10). Furthermore, BSA-Cu SAzymes have a good biosafety profile, making them a promising new treatment for colorectal cancer as they can be processed by the kidneys. Also relevant to the tumor microenvironment, probiotics may enable cancer biotherapy by secreting antitumor or immunomodulatory drugs in the tumor microenvironment. However, the efficacy and accuracy of probiotics in cancer treatment is limited, and the addition of nanozymes can ameliorate this shortcoming. Furthermore, studies on colon cancer both in vivo and in vitro have shown that the composite nanozyme system can efficiently suppress tumor growth via photothermally enhanced nano-catalytic synergistic therapies, offering a novel way to boost the effectiveness of chemodynamic therapy (CDT) [106, 107].

Liu et al.’s study [108] produced novel and effective Ir-N_5_ single-atom nanozymes for the treatment of liver tumors. These SAzymes mimic the enzyme cascade and disrupt the redox and metabolic homeostasis of the tumor region, producing an anticancer effect at the tumor site and amplifying oxidative stress by increasing ROS, which kills tumor cells with less impact on normal cells, resulting in an effective cancer therapy. One of the worst tumors is pancreatic cancer (PDAC), and using nanomaterials offers a fresh strategy to treat drug-resistant pancreatic. Currently, more experiments in the field of pancreatic cancer are carried out through the synergistic action of drugs or organic molecules with nanozymes to target delivery to the tumor site and reduce the drug resistance of cancer cells. Zhao et al. [109] synergized a metal-based SAzymes coupling (Fe@Fe_3_O_4_) with a naturally occurring biologically active organic molecule (ginseng saponin RG3) to construct a novel nanomedicine, which releases Fe^3+^ in the tumor TME and Fe^2+^ in the tumor TME and efficiently generates ROS thereby promoting cancer cell apoptosis. This also provides a new strategy for metal–organic nanocomposites to play a role in anticancer therapy.

Based on the cytochrome P450 structure, Sun et al. [110] created sandwich-like Sazymes and discovered that IrN_4_-S-TMN_4_ (TM = Co, Rh, Pd) showed selective CO_2_ reduction to CO with a special artificial CO dehydrogenase (CODH). They hypothesize that the unique enzyme-like activity and selectivity of SANs result from the precise control of sulfur atoms over the electron density, and that this control is contingent upon the nature and characteristics of the transition metals present in the surface layer. Feng et al. [111] immobilized ultramicro gold nanozymes in a metal–organic skeleton to establish a gold-iron SAzyme, which showed promising anti-esophageal cancer effects in vivo via the ferroptosis pathway. The same application of the ferroptosis pathway was also validated among the palladium nanozymes constructed by Zhang et al. [112]. Furthermore, an adaptive SAzymes-based iron death platform was constructed. This platform not only specifically enhances ROS generation activity, but also empowers SAzymes to consume GSH on demand in tumor cells, thereby accelerating selective and safe ferroptosis. The above anti-tumor responses have been demonstrated in colon cancers [96].

The limitations of traditional tumor treatment techniques have spawned the development of novel nanotechnology for the treatment of tumors. Currently, the application of nanozymes in gastrointestinal tumors is mainly limited to colorectal, liver and pancreatic cancers, and more studies are needed to explore their biological applications in other solid tumors (Table 2).

### Antioxidative therapy

Through chemical redox processes, antioxidant nanostructures can neutralize ROS. Numerous studies have suggested that by scavenging ROS and lowering the release of inflammatory factors, nanozymes can display superior anti-inflammatory and antioxidant benefits in the treatment of ulcerative colitis and inflammatory bowel disease (IBD) alone [9, 113]. Nevertheless, the contribution of biological SAzymes structures, which resemble natural enzymes in their activity, to the elimination of ROS remains incompletely understood.

ROS are scavenged by atomically dispersed Fe-N_4_ sites anchored on N-doped porous carbon materials (Fe-SAs/NC), which act as a bifunctional SAzyme for the removal of excess ROS produced during cellular oxidative stress. These sites mimic two antioxidant enzymes, CAT-like and SOD-like [91]. Comparable Fe-N/C monoatomic catalysts also exhibit glutathione-like, catalase-like, oxidase-like, and peroxidase-like activity. Intracellular H_2_O_2_ levels were successfully regulated by Fe-N/C SAzymes [114]. The findings of the two studies mentioned above demonstrated that the synthesized Fe-N/C SACs and Fe-SAs/NC were both more effective at scavenging the excess ROS that the oxidatively stressed cells produced. This suggests that the SAzymes could effectively shield the cells from the harm caused by cellular oxidative stress by scavenging the intracellular ROS, opening the door to the treatment of diseases linked to ROS. Not coincidentally, single-atom Pt/CeO_2_ treats brain injury by scavenging RONS by a mechanism that exhibits POD-like, CAT-like, and OXD-like multienzymatic catalytic activities [54]. Similar phenomena were found in an in vitro E. coli mouse model, which demonstrated the antioxidant activity of Co-SAzymes [89]. The CAT-like, SOD-like, and GSH-Px-like activities of Co-SAzymes reduced ROS and RNS in sepsis by 60–80%, and pro-inflammatory cytokine levels were reduced to 10% after 2 weeks of treatment. Because of its unique activity and kinetic features that are naturally similar to those of APX, graphitized carbon nitride (Cu SAs/CN) anchored with isolated single copper atoms can be employed to successfully protect H_2_O_2_-treated cells from oxidative damage in vitro [115]. Liu et al. [116] synthesized Pt@PCN222-Mn on the basis of manganese (III) porphyrin, which was doped with Mn-MOF to mimic superoxide dismutase and convert oxygen radicals into hydrogen peroxide. The experiments also mimicked the catalytic activity of catalase by doping platinum nanoparticles. Both in vitro and in vivo experimental measurements demonstrated the synergistic ROS scavenging ability of this integrated cascade of nanozymes.

Recent research has demonstrated that single-atom nanozymes, with their huge specific surface area, unusual electron packing, and superior electrical conductivity, possess intrinsic antioxidant characteristics. The application of nanozymes is a viable strategy to remove the excess intracellular ROS and to maintain the homeostasis of the cellular redox system because, despite the presence of several antioxidant natural enzymes such as CAT, SOD, and GSH-Px in the cellular system, the overexpression of ROS induced by pathological conditions inhibits the activity of the natural enzymes [117, 118] (Table 2).

### Biological detection

Excess GSH is a cancer biomarker that is crucial for preserving intracellular redox balance in tumor cells. Hemoglobin (Hb) as a Fe source embedded in zeolite imidazolate framework (ZIF-8) was utilized by Chen et al. [119] to create porous single-atom Fe nanozymes (pFe SAzymes) in a convenient and large-scale method, and then developed a pFe SAzymes-GSH assay, which can accurately detect GSH levels in tumor cells transplanted from liver in situ. The pFe SAzymes-GSH assay can detect GSH levels in liver in situ transplanted tumor cells with millimolar accuracy and avoids the need for complex manipulation, making it a simple, fast and accurate visualization method for identifying tumor boundaries. Catalytic nanomaterials called iron SAzymes (Fe-N-C SAzymes) were also made, and it was discovered that adenine and thymine exhibited a greater adsorption affinity on Fe-N-C SAzymes. Sun et al. [120] designed Apt/Fe-N-C SAzymes for the colorimetric assay of cancer cells based on the observation that one DNA sequence (adenine) in duplex DNA binds to Fe-N-C SAzymes and the other DNA sequence (i.e., aptamer) binds to cancer cells. This discovery offers a novel application of SAzymes in biomedicine. Nanozymes have also been used to distinguish between normal and cancerous cells for early identification of cancer. SAzymes has been utilized in investigations for the detection of various blood indicators in addition to tumor-related diagnoses (Table 2). In order to enable practical uric acid (UA) monitoring in serum samples, Hu et al. [121] investigated an A-Co-NG single-atom catalyst for electrochemical UA detection for the first time. They did this by attaching high-density and isolated cobalt atoms on an N-doped graphene substrate. Fe-SAzyme, which was produced by Zhou et al. [122], has a built-in colorimetric assay for galactose measurement and can be utilized as a substitute approach for diagnosing galactosemia. A flow-injection chemiluminescence immunoassay was created for the quick and accurate detection of serum 5-fluorouracil (5-Fu) in serum [123] based on the validation of the Fenton-like activity of Co-SAzymes.

## Discussion and perspectives

Among the deadliest cancers, gastrointestinal cancers cause around one-third of cancer-related deaths globally [124]. A number of gastrointestinal and liver conditions, such as inflammatory bowel disease (IBD) [125], colorectal cancer (CRC) [126], and alcohol-associated liver disease [127], have been related to alterations in the human gut microbiota. When inflammation reaches advanced stages, it can result in multiple organ dysfunction syndrome (MODS), infectious necrosis, and systemic inflammatory response syndrome (SIRS). One such condition is pancreatitis, which has a high morbidity and mortality rate and can be fatal [128]. Even though the digestive system is now developing quickly, new technologies are still required to solve current issues due to the diversity and complexity of disorders of the digestive tract, which places more demands on early diagnosis and accurate treatment.

Environmental protection, antibacterial, anticancer, and sensing are just a few of the many applications for SAzymes. Rethinking the connection between the structure of SAzymes and their active roles has proven difficult in light of the substantial changes that have occurred recently in the content, structure, and morphology of these molecules. Enzymes possessing atomically distributed metal active sites, as well as the ability to bind any ligand on appropriate carriers, exhibit significant promise for medicinal uses. This work establishes the groundwork for a later discussion of the function of SAzymes in gastrointestinal disorders by reviewing the most recent design principles and the preparation procedure of SAzymes, as well as by classifying and elaborating the biomimetic activities.

In addition, the complex internal environment and immune microenvironment in the body may affect the efficacy of SAzymes. The future research of SAzymes strategy is suggested by the significant number of vacancies in their study of inflammatory digestive system disorders such pancreatitis and cholecystitis. Currently, clinical investigations of SAzymes are not met, mostly due to the uncertainty over their biosafety. The ability of single-atom nanozymes to withstand degradation is one of the more significant variables. Despite numerous animal investigations have demonstrated that many SAzymes do not have harmful effects on other organs, the risks associated with a continuous presence of these enzymes in the bloodstream remain unclear, particularly with regard to critical organs like the heart and brain [129]. Furthermore, since different SAzymes have different active centers and framework structures, toxicity occurs when their structural composition, elemental loading composition, internal environment, and mode of administration are changed. Future research should focus on improving the in vivo biostability of single-atom nanozymes and clarifying the scope of their uses in microbial bioassay, targeted drug delivery, immune regulation and anti-inflammatory therapy [130, 131]. A thorough safety evaluation that takes into account of pharmacokinetics, body organ distribution, and in vivo metabolism is still necessary for the clinical use of SAzymes.

## Data Availability

Not applicable.
